# Measurement of ionized calcium using the Nova 2 analyser

**DOI:** 10.1155/S1463924683000127

**Published:** 1983

**Authors:** P. Ferreira, B. E. Jacobson

**Affiliations:** 1 Department of Pathotogy Division of Clinical Chemistry Vancouver General Hospital British Columbia Vancouver V57 IMP Canada; 2 Department of Pediatrics University of Alberta Hospital Alberta Edmonton T6G 2B7 Canada


Journal of Automatic Chemistry, Volume 5, Number 1 (January–March 1983), pages 40–42

### Measurement of ionized calcium using the Nova 2 analyser

**P. Ferreira*** **and B. E. Jacobson**

*Department of Pathology, Division of Clinical Chemistry, Vancouver General Hospital, Vancouver, British Columbia V57 IM9, Canada*

### Introduction

Approximately 50% of plasma calcium is present as the physiologically active 'ionized' or 'free' form. Although routine clinical assessment of this fraction is usually made from knowledge of the total calcium, total protein and albumin, there is increasing evidence that in pathological situations this approach does not correlate well with the direct measurement of ionized calcium [1].

This study was undertaken to investigate the performance of a new ionized calcium analyser, the 'Nova 2', and determine its practicability in the routine clinical laboratory.

### Materials and methods

The Nova 2 analyser (Nova Biomedical, Newton, Massachusetts, USA) is an automatic instrument that measures ionized calcium by direct potentiometry. It was used according to the manufacturer's instructions and with the reagents and standards supplied. The data reported here was gathered over a period of five months. Because of the unavailability of a commercial control serum for ionized calcium, one was prepared in the following way: pooled serum was collected from normal individuals and first allowed to equilibrate with the atmosphere, then buffered with 10 mmol/l Tris/HCl at pH 7·4. The total calcium was 2·3 mmol/l (Control A).

A second pool was prepared in a similar way, but $CaCl_2$ was added to yield a total calcium of 3·3 mmol/l (Control B). These control sera were aliquoted and stored deep frozen at −80°C.

Total calcium and total protein were measured on a Dupont ACA (Wilmington, Delaware, USA) and an Abbott ABA-100 (Chicago, Illinois, USA) respectively. Albumin was quantitated by electrophoresis on cellulose acetate [2]. Corrected calcium (in mmol/l) was calculated as follows:

(A) *Total protein correction* [3]:

$$\text{Corrected calcium} = \frac{\text{measured calcium}}{\left[0{\cdot}6 + \dfrac{0{\cdot}4 \times \text{TP (g/l)}}{74}\right]}$$

(B) *Albumin correction* [4]:

$$\text{Corrected calcium} = \text{measured calcium} + 0{\cdot}025[\text{albumin (g/l)} - 40].$$

In all experiments involving serum, plasma or whole blood samples, strict anaerobiasis was maintained, either by direct sampling from vacutainers, or storage of samples in capped syringes with direct introduction into the analyser probe using a short length of plastic tubing. Samples from patients undergoing plasmapheresis were taken immediately before and after the procedure itself.

### Results

### *Precision*

When replicate serum analyses were performed sequentially the results progressively decreased. This effect could be prevented by preceding each serum analysis with an aqueous standard. This effect was not appreciated until about one-third of the way through the project, which could partly explain why the control values up to this point tended to be lower than those recorded later. It would also explain the improved precision after this time when the above procedure was carried out routinely. (All patients' results reported in this study were preceded by aqueous standards.) Precision improved when serum or plasma was used rather than whole blood on patient samples; however, precision was worse using control serum than with patient samples (table 1).

### *Accuracy*

*Sampling methods*

The analyser is designed to sample anaerobically whole blood, plasma, or serum directly from a vacutainer or syringe. However, the analyser tubing is made from silicone rubber which is carbon dioxide-permeable and therefore could allow significant $CO_2$ loss and consequent pH and ionized calcium changes [5]. This was demonstrated by replacing the tubing with $CO_2$ impermeable material (polypropylene). The result was a slight, but significant, rise in the mean of results by 0·015 mmol/l ($p < 0{\cdot}01$).

*Table 1. Precision.*

| | *N* | Mean (mmol/l) | SD (mmol/l) | CV (%) |
|---|---|---|---|---|
| (*a*) *Within-run* | | | | |
| *Aqueous standards:* | | | | |
| 0·5 mmol/l | 21 | 0·492 | 0·004 | 0·87 |
| 1·5 mmol/l | 46 | 1·502 | 0·009 | 0·57 |
| 2·5 mmol/l | 19 | 2·557 | 0·017 | 0·65 |
| Control sera: A | 17 | 1·330 | 0·016 | 1·22 |
| B | 17 | 1·958 | 0·039 | 2·00 |
| *Patient samples:* | | | | |
| Whole blood | 17 | 1·183 | 0·014 | 1·18 |
| Plasma | 12 | 1·120 | 0·003 | 0·23 |
| Duplicate sera | 64 | 1·150 | 0·007 | 0·62 |
| (*b*) *Between-run* | | | | |
| *All data:* | | | | |
| Control A | 56 | 1·345 | 0·023 | 1·71 |
| Control B | 57 | 1·997 | 0·046 | 2·30 |
| *Later data only**: | | | | |
| Control A | 34 | 1·357 | 0·014 | 1·03 |
| Control B | 35 | 2·017 | 0·029 | 1·44 |

* After the policy was instituted of preceding each serum analysis with an aqueous standard.

* *Present address: Department of Pediatrics, University of Alberta Hospital, Edmonton, Alberta T6G 2B7, Canada.*

*Whole blood versus plasma*

This was investigated on five separate occasions by running replicate analyses on whole blood and anaerobically-separated plasma from the same individual. The results were inconsistent; on two days there was less than 0·010 mmol/l difference ($p>0·05$), but on three other occasions the results were higher on the whole blood samples by 0·070 mmol/l ($p<0·01$), 0·066 mmol/l ($p<0·001$), and 0·086 mmol/l ($p<0·001$).

*Serum versus plasma*

No statistically significant difference could be found between results on serum and plasma when each was sampled from standard vacutainers. (Heparin concentration of plasma approximately 25 units/ml.) However, a higher concentration of heparin, typically that used in blood gas analysis (approximately 200–400 units per ml), was found to depress results by a mean of 8% ($p<0·001$). It is important to fill vacutainers completely; vacutainers which were only half full gave results on average 3% lower than full ones.

*Effect of changing electrodes*

Fifteen patient sera were run on each of two electrodes. A marked difference in results were obtained, despite successful aqueous calibration in each case (table 2). Care was taken to keep the samples anaerobic and to avoid any timing bias: half the samples were run on electrode A, the electrodes were switched, all were run on B, then the other half run on A.

*Table 2. Effect of changing electrode on ionized calcium results.*

| | Mean* (mmol/l) | SD (mmol/l) | |
|---|---|---|---|
| Electrode A | 1·272 | 0·236 | |
| Electrode B | 1·199 | 0·237 | |
| Difference (A–B) | 0·072 | 0·009 | ($p=<0001$) |

*$N=15$ patient sera.

*Normal range*

This was established on venous plasma samples of 17 normal individuals of both sexes and aged between 18 and 40. Mean 1·204 mmol/l, SD 0·047 mmol/l; mean ± 2 SD = 1·11 0 – 1·298 mmol/l.

*Ionic interferences*

Interference was tested on aqueous calcium chloride solutions that were buffered with 5 mmol/l Tris-HCl (pH 7·4 except in pH studies). Results were plotted against several concentrations of added ions and mean interferences calculated from the regression line slopes. All plots were reasonably linear. The data are summarized in table 3.

*Protein interference*

Four patient sera were anaerobically ultrafiltered at 37°C using Worthington 'Ultrafree' filters. When analysed using the Nova 2, the apparent ionized calcium was lower in the ultrafiltrate, and higher in the protein-rich serum remaining after ultrafiltration, with the native serum results in between (figure 1). There was also a significant correlation observed between ionized calcium and total protein ($r=0·36$, $p<0·001$) in a group of 78 hospital patients with miscellaneous complaints. The regression line slope was 0·0049 mmol/l of ionized calcium per g/l protein.

*Table 3. Ionic interferences.*

| Interfering ion | Concentration range studied | Mean interference |
|---|---|---|
| | | (Apparent change in ionized calcium) |
| pH | 6·77–8·00 | −0·007 mmol/l ionized calcium per 0·1 pH unit increase |
| Mg++ | 1–5 mmol/l | −0·066 mmol/l ionized calcium per 1 mmol/l Mg++ increase |
| $Na^+$ | 100–175 mmol/l | −0·012 mmol/l ionized calcium per 10 mmol/l Na+ increase |

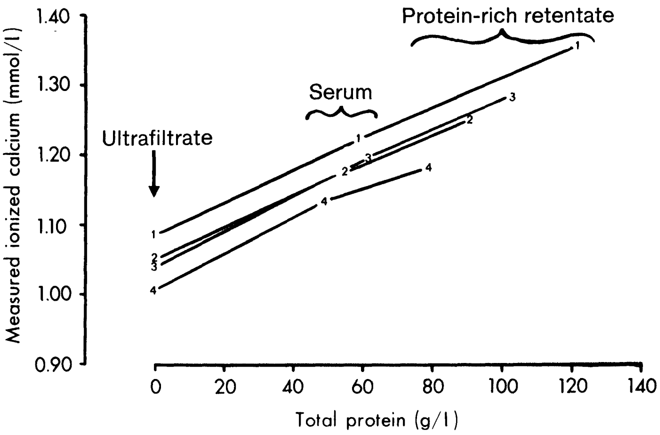

*Figure 1. Effect of protein concentration on apparent ionized calcium measured on the Nova 2 analyser. Four sera (1–4) were anaerobically ultrafiltered at 37°C to yield the fractions shown.*

*Comparison with total calcium results*

There is a good overall correlation ($r=0·89$) with total calcium in the patients studied (figure 2). Interestingly, the correlation coefficient is lower when the total calcium is corrected for albumin ($r=0·57$) or total protein ($r=0·66$).

Patients undergoing plasmapheresis all showed a consistent decrease in both ionized and total calcium, though the fall in the ionized fraction was proportionally greater (table 4).

*Reliability*

No major instrument problems developed during this evaluation. Preventative maintenance is well explained in the manufacturer's manual and easy to perform. Tubing tended to become blocked if left in the 'stand-by mode' for more than one day. This can be avoided by using the 'stat mode', which is rather expensive on reagents, or by calibrating the instrument at least twice daily in the 'stand-by mode'.

### Discussion

Within-run and between-run precision is acceptable, but it is recommended that all serum analysis be preceded by aqueous standards in order to achieve optimum performance. The reason for poorer precision using control serum rather than patient samples is not clear.

Whole blood samples give poorer precision and do not

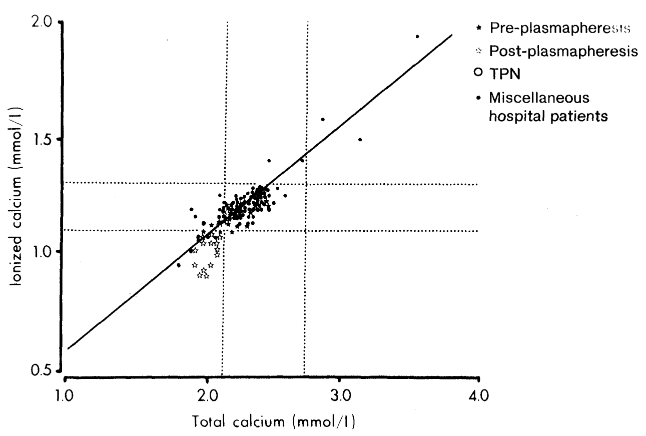

*Figure 2. 'Scattergram' of ionized calcium versus total calcium. Different groups of patients are identified. Enclosed horizontal and vertical lines represent normal range limits. Correlation coefficient (r) = 0·89.*

*Table 4. Ionized calcium in 17 plasmapheresis patients (mean ± SD).*

| | Pre | Post | Significance* |
|---|---|---|---|
| Ionized calcium (mmol/l) | 1·14 ± 0·09 | 0·99 ± 0·08 | $p<0·001$ |
| Total calcium (mmol/l) | 2·21 ± 0·12 | 1·99 ± 0·10 | $p<0·001$ |
| Albumin corrected total calcium (mmol/l) | 2·27 ± 0·14 | 2·03 ± 0·09 | $p<0·001$ |
| Total protein corrected total calcium (mmol/l) | 2·43 ± 0·09 | 2·37 ± 0·09 | $p=0·04$ |
| Total protein (g/l) | 58·4 ± 8·6 | 44·6 ± 4·2 | $p<0·001$ |
| Albumin (g/l) | 38·0 ± 5·5 | 38·1 ± 2·5 | N.S. |

* Paired Student's t-test.

correlate well with plasma results. Most previous authors using both the Orion SS-20 [5–7] and the Nova 2 [8] have found increased values in whole blood, but in one study the difference was minimal [9]. Increased values could be explained by red cells altering the potential at the liquid junction [5 and 7]. Exactly why the results in this study were variable from day to day is difficult to explain. The liquid junction in the Nova 2 is situated within the reference electrode, and it is possible that build-up of potassium chloride crystals might alter the characteristics of the liquid junction from day to day, without blocking the flow completely.

Serum protein may also alter the residual junction potential, and has been reported to cause positive interference in the measurement of ionized calcium [5 and 7]. This study suggests a similar problem: when protein was separated by ultrafiltration, the apparent ionized calcium fell more than the approximately 5% anticipated from the Donnan effect [10]. Also, the correlation observed between ionized calcium and protein levels in patient samples tends to confirm this hypothesis. Ionic interferences with the exception of magnesium are minimal. The discrepancy between the negative interference reported here and the positive interference reported by Fyffe *et al.* [8] is difficult to explain unless individual electrodes vary widely in their responses. There appears to be a negative bias introduced by the carbon dioxide permeability of silicone rubber tubing; however, this is likely to be fairly constant and its minor extent makes it of little clinical importance. A more important source of error is the variability in results between electrodes, which has also been reported in previous studies of the Nova [8], as well as the Orion SS-20 [5]. If this is a regular and expected occurrence, then the normal range would have to be checked and possibly changed with each new electrode that was used, unless it could be shown that the difference in control serum values accurately parallelled those of patient samples and results are corrected accordingly [5].

In the patients studied, ionized calcium levels showed a good correlation with total calcium, similar to previous published studies [11]. In theory, one would expect the correlation to improve if total calcium is corrected for albumin or total protein levels. The fact that this does not happen is consistent with other reports that 'correction factors' do not actually work well in practice [1].

Patients undergoing plasmapheresis show a marked drop in ionized and total calcium, with a proportionally greater fall in the former. This is similar to the results reported by Morse *et al.* [12]. A few patients experienced symptoms of hypocalcemia, such as circumoral paresthesia. This probably related to the use of citrate as an anticoagulant which complexes ionic calcium. However, the low protein level post-plasmapheresis could also lead to artifactually low measured ionized calcium levels (c.f. protein effect, above). Patients on total parenteral nutrition have unremarkable ionized calcium concentration, being either within or near the normal range.

### Conclusion

Generally, this is a very simple instrument to operate, which gives reproducible results given a few simple precautions. Direct serum sampling from vacutainers is a very convenient way to introduce anaerobic samples. This instrument is a definite advance over previous instruments and should make routine measurement of ionized calcium more readily available for the clinical laboratory.

### Acknowledgement

The authors are grateful to Fisher Scientific Ltd for the loan of the Nova 2 analyser used in this evaluation.

### References

1. Ladenson, J. H., Lewis, J. W. and Boyd, J. C., *Journal of Clinical Endocrinology and Metabolism*, **46** (1978), 986.

2. Grumbaum, B. W., Zec, J. and Durrum, E. I., *Microchemistry*, **7** (1963), 41.

3. Parfitt, A M., *New England Journal of Medicine*, **281** (1969), 55.

4. Payne, R. B., Little, A. J., Williams, R. B. and Milner, J. R., *British Medical Journal*, **4** (1973), 643.

5. Ferreira, P. and Bold, A. M., *Journal of Automatic Chemistry*, **1** (1979), 94.

6. Masden, S. and Olgaard, K., *Clinical Chemistry*, **23** (1977), 690.

7. Larson, L. and Ohman, S., *Clinical Chemistry*, **24** (1978), 731.

8. Fyffe, J. A., Jenkins, A. S., Cohen, H. N., Dryburgh, F. J. and Gardner, M. D., *Journal of Automatic Chemistry*, **2** (1980), 85.

9. Fuchs, C., Dorn, D., McIntosh, L. and Scheler, F., *Clinica Chemica Acta*, **67** (1976), 99.

10. Rose, G. A., *Clinica Chemica Acta*, **2** (1957), 227.

11. Ladenson, J. H. and Bowers, G. N., *Clinical Chemistry*, **19** (1973), 575.

12. Morse, E. E., Hohnadel, D. C., Genco, P. and Katz, A. J., *Johns Hopkins Medical Journal*, **146** (1980), 260.

